# Spending by California’s Department of Developmental Services for Persons with Autism across Demographic and Expenditure Categories

**DOI:** 10.1371/journal.pone.0151970

**Published:** 2016-03-25

**Authors:** J. Paul Leigh, Scott D. Grosse, Diana Cassady, Joy Melnikow, Irva Hertz-Picciotto

**Affiliations:** 1 Center for Healthcare Policy and Research, University of California Davis School of Medicine, Sacramento, California, United States of America; 2 Department of Public Health Sciences, University of California Davis School of Medicine, Davis, California, United States of America; 3 National Center on Birth Defects and Developmental Disabilities, Centers for Disease Control and Prevention, Atlanta, GA, United States of America; 4 Department of Family Medicine, University of California Davis, School of Medicine, Sacramento, California, United States of America; UNC Chapel Hill, UNITED STATES

## Abstract

**Background:**

Few autism spectrum disorder (ASD) studies have estimated non-medical costs for treatment or addressed possible differences in provision of services across gender, race-ethnic, age or demographic or expenditure categories, especially among adults.

**Methods:**

The California Department of Developmental Services (CDDS) provides services to residents with developmental disabilities. CDDS provided aggregate data on primarily non-medical spending for fiscal year 2012–2013 for persons with ASD with or without intellectual disability (ID) (main sample, n = 42,274), and two sub-samples: ASD only (n = 30,164), and ASD+ID (n = 12,110). Demographic variables included sex, age and race-ethnicity. Spending categories included Employment Support, Community Care Facilities, Day Care, Transportation, and in-home and out-of-home Respite.

**Results:**

Per-person spending for males and females were approximately the same: $10,488 and $10,791 for males and females for ages 3–17 and $26,491 and $26,627 for ages 18+. Among race/ethnicity categories, the ranking from highest to lowest among ages 3–17 was white non-Hispanics ($11,480), Asian non-Hispanics ($11,036), “Others” ($11,031), Hispanics ($9,571), and African-American non-Hispanics ($9,482). For ages 18+, the ranking was whites ($31,008), African-Americans ($26,831), “Others” ($25,395), Asians ($22,993), and Hispanics ($18,083). The ASD+ID sub-sample exerted disproportionate influence on findings from the main sample for persons 18+. Combining all ages, the top two expenditure categories for per-person spending were Community Care Facilities ($43,867) and Day Care ($11,244). For most adult age groups, the percentage of recipients participating were highest for Day Care (44.9% - 62.4%) and Transportation (38.6% - 50.9%). Per-person spending for Day Care, Transportation, and Employment Support was relatively low for children but relatively high for adults.

**Conclusion:**

White non-Hispanics received the highest per-person spending and Hispanics among the least. Amounts within spending categories varied considerably across age groups. Our estimates may be useful as baseline measures for stakeholders preparing for increasing ASD prevalence, especially among adults.

## Introduction

The reported prevalence of diagnoses of Autism Spectrum Disorders (ASD) has increased rapidly, from approximately 1 in 150 8-year-old American children in 2000 to 1 in 68 in 2010[1]. Although part of the increase in prevalence appears due to broadened diagnostic criteria, and a downward shift is expected to occur with the introduction of new DSM-5 criteria[2], there is concern that the increase in the demand for medical, behavioral, residential, occupational, and other services for children and young adults with ASD diagnoses may be outstripping the supply of services[3].

Disease costs for ASD are grouped into three categories: direct medical, direct non-medical, and indirect productivity costs[4]. All three categories are substantial, but what sets ASD apart from many other diseases and conditions are the direct non-medical costs. These non-medical costs include: educational and transportation assistance; behavioral, occupational, and speech therapy; and family caregiver time. Estimates of costs for many diseases such as heart disease, cancer, stroke, and hypertension frequently are limited to medical and productivity costs[5,6]. But relying on medical and productivity costs alone for estimating costs of ASD is a seriously flawed approach. Non-medical costs for children, youths, and adults with ASD are much larger than medical costs[4,7].

It is not clear how many hours per-week of non-medical intervention is required for optimal improvement among children with ASD[8]. The American Academy of Pediatrics Consensus Guidelines for non-medical interventions for children with ASD recommends that “children should have access to at least 25 hours per week of comprehensive intervention to address social, communication, language, play skills, and maladaptive behavior”[9].”This 25 hours guideline, however, was endorsed as “strong” by only 56% of the Technical Expert Panel, the lowest rating of any of the guidelines. Moreover, the Early Start Denver Model involves only 15 hours per-week[10].

Whereas our study focused on differences in spending on services across demographic and expenditure categories, related studies have focused on the extent and type of unmet need. Hodgetts et al (2015)[11] recent study of 143 children with ASD in Alberta, Canada indicated that “overall”, in their sample, “families…had many needs relatively well met”[11]. Hodgetts et al (2015)[11] also found that the extent of unmet needs, if any, varied across groups. For example, parents with high income and younger children reported few or no unmet needs but parents with low income and older children reported significant unmet needs. Hodgetts et al (2015)[11]found that respite care was the highest ranking unmet need of all services considered. A separate Canadian study of 101 families found significant unmet needs for “social activities for my child” but far fewer unmet needs for “physical therapy for my child”[12]. Some US studies indicate that a significant minority (33–43%) of young children with ASD receive no regular behavioral intervention[13,14].

Our study uses data on expenditures incurred by the California Department of Developmental Services (CDDS), the state agency that administers state mandated programs to provide or coordinate services to adults, children, and parents of children with developmental disabilities[15]. CDDS data are compiled from quarterly Client Development Evaluation Reports filed by 21 regional centers, and include both medical (ICD-9/ICD-10 codes) and psychiatric (DSM-4/DSM-5 codes) diagnosis codes as well assessments performed by the regional centers with which CDDS contracts to provide services. CDDS data have been used extensively for ASD research as well as for research on the economics of childhood disabilities[16–20]. For example, a recent study on diagnostic substitution estimated that for every four new ASD cases enrolled by CDDS, one fewer new case of mild intellectual disability was enrolled[21]. Whereas other states provide services for ASD patients and families through Medicaid, in California CDDS has the explicit mission to provide services in addition to those provided by Medicaid (MediCal in California). CDDS has been estimated to provide services to 75%-80% of persons diagnosed with ASD[22,23]. Despite the extensive detail in the CDDS data, we are not aware of previously published studies in the scientific literature that report estimates of annual spending per person on cohorts with ASD. We provide these estimates and, more importantly, compare spending per person across gender, age, and race/ethnic categories

Among studies reporting health care costs for children and youth with ASD, a few have reported mean spending per person separately for males and females. The male-female difference in per-person expenditures has been reported to be relatively small in magnitude and not statistically significant[24–26]. Although race/ethnicity information is typically lacking in insurance claims databases, in a nationwide sample of Medicaid-enrolled children with ASD diagnoses in 2005, average Medicaid expenditures per person for white children were higher by $1693 than for nonwhite children, or 15.8% more than the mean for white and nonwhite combined[24]. In a small UK sample of adolescents with ASD, mean total costs, which were primarily educational, were 40.9% higher for white than non-white youth[26].Among children ages 24–60 months, mean costs were 10.0% higher for white than non-white children[26].

The literature on ASD cost differences across age groups is relatively sparse and with varying findings. Cidav et al.[27] analyzed Medicaid data in 2005 and found that spending increased about 5% for each year from age 3 to age 20. Schlenz et al.[28], on the other hand, did not find significant differences in utilization of hospital services for psychiatric conditions in preadolescents (ages 9–12) versus adolescents (13–18) in South Carolina. Two UK studies examined differences by age in costs for children with ASD. Among children ages 24–60 months, total costs increased significantly with age[25], whereas among adolescents ages 14–17 years, the younger half of the sample had significantly higher mean costs[26].

A binational modeling study by Buescher et al.[29] compared projected per-person costs in the United Kingdom and the United States; the UK cost estimates drew on an earlier publication by Knapp et al.[30]. Buescher et al.[29] assumed that per-person costs for special education are much higher for children ages 0–5 with ASD in the United States than for children ages 6–17 whereas in the United Kingdom special education was assumed to be most costly at ages 12–17 and lowest at ages 0–3. Accommodation or residential care costs and medical costs were assumed to be higher for older children or adolescents in both countries. The cost of nonmedical services was assumed to peak at ages 4–11 for UK children and to be invariant with age for US children.

We are not aware of studies on the costs of services that directly compare adults with children or adolescents using the same data set. The few studies on adults nevertheless tend to produce estimates that are larger than those for children using separate data sets[4]. For example, Lakin et al.[31] reported Medicaid annual spending of $128,300 for adults with ID and ASD, a number that far exceeded any other annual figure for spending on children or adolescents in the Amendah et al.[4] comprehensive review.

Among studies on developmental costs, few have addressed how spending varied across more than two to four categories. The most prominent exception was the study by Cidav et al.[27] who used national data on children and youths enrolled in Medicaid in 2005. Cidav et al [27] analyzed spending patterns within 14 categories and across four age groups (3–6, 7–11, 12–16, and 17–20). Increasing age was associated with higher spending for long-term care, psychiatric medications, case management, partial hospitalizations, and respite services but with lower spending on occupational and physical therapy, speech therapy, mental health services, diagnostic services, and family therapy.

Our study addresses sex, race/ethnicity, and age differences in expenditures for developmental services that are primarily non-medical but a few are medical. For ease of presentation, we refer to these services combined as “predominantly non-medical.” We also address differences in eight spending categories as defined by the CDDS. We have several motivations. First, few US studies have reported estimates of per-person non-medical expenditures for services to persons with developmental disabilities. Second, we are not aware of any predominantly non-medical ASD expenditure study that focused on demographic differences, including race/ethnic differences. Third, we are not aware of any studies that focus on developmental categories of spending across adult age groups. Finally, there is controversy surrounding the proper role of state governments in providing services for persons with ASD[32]. Our study documents the per-person dollar amount of services provided by the state of California for fiscal year 2013. For the eight categories of spending, we also document the total spending, percentage of recipients participating, and average per-person spending for those with non-zero spending. Results from this study may also be of value for policy makers and others who have a need to plan for future needs, given the growing number of children with ASD and the projected increases in adults with the condition.

## Method

The CDDS defines developmental disabilities to include intellectual disability (mental retardation), epilepsy, cerebral palsy, autism, and other conditions[15]. The CDDS provides services to individuals and families and carries out its mission through 21 regional and statewide centers. While CDDS is the most comprehensive record of non-medical service receipt for children and adults with developmental disabilities in California, not everyone with disabilities is served. Some people never apply for services and others do not meet CDDS eligibility criteria. Personnel at the 21 regional centers establish eligibility using medical (ICD-9 and ICD-10 codes) and psychiatric (DSM-4 and DSM-5 codes) diagnoses. To meet the CDDS eligibility criteria, the disability must have begun before the person’s 18th birthday and be expected to continue indefinitely. The disability must be “substantial” as defined by section 4512 of the California Welfare and Institutions Code[33].

The CDDS defines its services within 10 categories. For the most recent fiscal year of published data, 2007, the categories (and percent of funds) were: Day Programs (25.3% of total spending), Out-of-Home (25.0%), Support Services (17.5%), Miscellaneous Services (9.0%), Transportation (7.3%), In-Home Respite (6.2%), Supported Employment (3.0%), Health Care (2.7%), Work Activity Program (2.4%) and Out-of-Home Respite (1.7%) (sums to 100.1% due to rounding)[34]. “Day Programs” involve training in behavior management, self-help skills, and development programs for infants. “Out-of-Home” includes the care, supervision, and training for people in community care facilities. “Transportation” includes buses, trains, and vehicle travel with care-taking personnel, family or friends. “Supported Employment Programs” pay for job coaches to help subjects complete job-tasks at their place of business. “Work Activity Programs” are for work-related services, including vocational training, provided to subjects who are paid for their work.

Following Ganz[35], we acknowledge that sometimes the distinction between medical and non-medical costs can be blurred as, for example, when behavioral therapy is included under medical costs. We therefore have included the CDDS category “Health Care” in our analysis of demographic differences even though it comprises only 2.7% of total CDDS spending. This expenditure is a small percentage of total medical spending on ASD, most of which is paid by private insurance carriers, MediCal, Medicare, and individuals and families (personal communication with Elizabeth Hibbert, Privacy Officer, California Department of Developmental Services, Information Services Division, May 25, 2011). In the analysis of the eight categories of spending, however, we excluded the CDDS category for “Health Care.” Attempts to generalize about all medical spending based on these limited CDDS data would be problematic.

The CDDS budget was approximately $5.0 billion for fiscal year 2013–2014[36]. The CDDS data contain information on spending, age, gender, and race/ethnicity among other variables. CDDS provided us with the latest data available in December 2013 which contained 99.8% of all the information for fiscal year 2013. (S1 Dataset; S2 Dataset). CDDS refers to recipients of services as “customers” or “clients”; we will use “subjects” or “persons.” We provide the numbers of persons, annual mean spending per person, and standard deviations of spending per person. Demographic categories included gender, race/ethnicity (white non-Hispanic, African-American non-Hispanic, Asian non-Hispanic, Hispanic, and a category we created, “other” non-Hispanic), and age in years (3–6, 7–11, 12–16, 17–20, 21–24, 25–34, 35–44, 45–54, 55–64, and 65+). “Other” includes non-responders, Native Americans and Pacific Islanders. These age categories corresponded to those used by Cidav et al.[27] who also excluded ages 0–2 due to concerns about the validity of diagnosis.

We initially sought to measure spending associated with ASD stratified by co-occurrence with other disabilities. Sizable percentages—from 25% to 70%—of persons with ASD have been reported to also have intellectual disability (ID)[37].However, in the latest CDDS Fact Book[34], subjects with ASD accounted for 17.7% of all CDDS subjects, including 5.2% with both ASD and ID diagnoses and 12.5% with a recorded diagnosis only for ASD; almost one-half (46.1%) of all persons served had ID only (i.e., no ASD or cerebral palsy or epilepsy diagnosis.) These data suggest that just 29.4% of CDDS subjects with ASD had the combination of ASD and ID. This 29.4% contrasts sharply with findings in the literature in which children with ASD were given cognitive tests. Given the requirements for substantial functional impairments to receive services through the DDS, this figure suggests possible under-diagnosis of ID among persons with ASD in this database. Fombonne[38] estimates that roughly 40% of patients with ASD also have ID. Many previous studies included persons with the combination of ASD and ID[4,27,29,34,39]. Accordingly, in our main analysis, we reported annual mean spending per person for individuals with ASD with or without the additional diagnosis in the CDDS record of intellectual disability, while acknowledging that this distinction may be subject to misclassification. (S1 Dataset). In a secondary analysis, we separated data into sub-sets: those with ASD only and those with both ASD and ID (ASD+ID). (S2 Dataset).

In the latest CDDS Fact Book, combinations of ASD with either cerebral palsy or epilepsy were uncommon, comprising less than one-half of one percent of subjects[34]. It is likely that CDDS data underestimate co-occurrence of ASD with epilepsy. Jester and Tuchman [40] review of the literature suggests 6 to 27% of persons with an ASD diagnosis also have epilepsy. We excluded the one-half of one percent of CDDS subjects with ASD and either cerebral palsy or epilepsy.

For spending data, we reported mean expenditures for fiscal year 2013 and also displayed data in box and whiskers diagrams. We took two approaches to analyze mean differences: descriptive and hypothesis-testing. In the descriptive approach, we recognized that we had the entire universe (population) of data for fiscal year 2013. This descriptive approach did not require hypothesis tests but simply judgment on the magnitude of differences[41]. The second approach assumed that the 2013 fiscal year dataset was a random sample for the most recent years of CDDS data. This second approach compared means with z-scores using the usual formula for the standard error for the difference in means of continuous variables drawn from different populations[41]. We prefer this second approach as it accounts for small sample sizes in some comparisons. We report statistical tests of significance at the 0.01 and 0.05 levels; unless otherwise stated, statistically significant differences are significant at the 0.05 level.

Because spending is likely to vary across age groups, our initial analysis stratifies data into two age groups: children and adolescents (ages 3–17) and adults (18+). Our second analysis uses 10 age groups: 3–6, 7–11, 12–16, 17–20, 21–24, 25–34, 35–44, 45–54, 55–64, and 65+. For numbers of subjects, we estimated CDDS-specific service prevalence rates by age group. Denominators were estimates of the California population for each age using data from the California Department of Finance (2013). CDDS-specific prevalence of receipt of developmental services was measured as per-1000 population within age groups.

Table 1 presents descriptions of eight CDDS categories of spending. Our first category combined three of the original CDDS categories: group employment support, individual employment support, and work activity programs and we labeled it Employment Support. All three applied to work and each, individually, involved a small amount of funds. The final two CDDS categories were Support Services (and included 18 separate types of services) and Miscellaneous (and included over 100 separate types of services). CDDS did not provide us with separate spending data on these 118+ types, however. In the analysis that follows, we chose to de-emphasize information on Support Services and Miscellaneous for two reasons. First, the general categories of Support Services and Miscellaneous are not particularly informative. Second, Support Services and Miscellaneous include some types of spending such as adaptive skills training, behavior management, and creative arts that would also likely be provided by public schools. Total state government spending within Support Services and Miscellaneous would therefore be underestimated, perhaps by a significant margin. This criticism would likely not apply to some of the remaining categories such as Employment Support, In-home Respite, and Out-of-home Respite. Moreover, this criticism would not apply to adults.

**Table 1:** Description of Categories of Spending.

Category	Description
Supplemented employment–group; Supplemented employment–individual; Work Activity programs	Individual and group services in integrated settings where paid workers are supported by job coaches, rehabilitative work services and vocational training.
Community Care Facilities	Community Care Facilities and out-of-home services.
Day Care Programs	Includes community-based training such as behavior management, self-help and self-care skills, community integration, and infant development programs.
Transportation	Transportation for subject and for care-giving personnel. by Transportation companies, buses, trains, and vehicles, residential facilities, day programs, public Transportation, and family and friends.
In-home Respite	Short-term care provided by paid caregiver in the home to allow usual family caregiver(s) a short break. Paid caregiver may: ensure medicine is appropriately administered; ensure patient attends scheduled therapy sessions; cook; clean; and so on.
Out of home respite	Short-term care provided within licensed facilities to allow usual family caregiver(s) a short break. Caregivers within facilities may: ensure medicine is appropriately administered; ensure patient attends scheduled therapy sessions; provide meals; provide private rooms for overnight stays; and so on.
Support Services	Support Services lists 18 categories of spending. Here are some: crisis evaluation and behavioral intervention; personal emergency response system; community integration training program; parent coordinated support living program; supplemental day services; supplemental program support; adaptive skills trainer; behavior management consultant; home health agency; and supported living series vendor administration
Miscellaneous	Includes over 100 separate categories such as: translator; out-of-state manufacturer or distributor; diaper and nutritional voucher s; special Olympics; funeral services; foster grandparent program; sports club; school for the deaf and blind; money management; out-of-state residential treatment; public school early intervention program; creative arts; and attorney fees.

## Results

There are three sub-sections within this Results section. We first present demographic differences within the sample comprised of persons with ASD who may or may not also have ID. The second sub-section analyzes the same demographic differences for two different sub-samples: persons with ASD only; and persons with ASD and ID. The third sub-section presents results on the eight expenditure categories with data from the larger, main sample.

### Persons with ASD with or without ID (Main Sample)

Table 2 presents spending data for males and females for those with ASD with or without ID. The top three rows present the overall number of subjects, mean spending per-person by CDDS, and standard deviation. The bottom five rows present data on differences in mean spending across categories. We found nearly three times as many males as females with ASD (26,174 male and 8758 female for ages 3–17; 5343 male and 1999 female for ages 18+)

**Table 2:** Gender, number of subjects, means and differences for spending. N = 42,274.

Categories	Males ages 3–17	Males, ages 18+	Females ages 3–17	Females, ages 18+
Number of subjects	26,174	8,758	5,343	1,999
Mean spending	$10,488	$26,491	$10,791	$26,627
Standard deviation	$14,159	$37,037	$15,445	$36,537
Mean differences and p-values subtracting column value minus row value				
Males, ages 18+	$10,488			
	-$26,491			
	= -$16,003			
	(p<0.0001)**			
Females, ages 3–17	$10,488	$26,491		
	-$10,791	-$10,791		
	= -$303	= $15,700		
	(p = 0.1852)	(p<0.0001)**		
Females, ages 18+	$10,488	$26,491	$10,791	
	-$26,627	-$26,627	-$26,627	
	= -$16,139	= -$136	= -$15,836	
	(p<0.0001)**	(p<0.8809)	(p<0.0001)**	

** Indicates statistical significance at the 0.01 level.

CDDS spent approximately the same for males and females within the same age group (Table 2). Slightly more was spent on females: $303 (p = 0.1852) (or 2.9% above the male mean) for ages 3–17 and $163 (p = 0.8809) (or 0.5% above the male mean) for ages 18+.

**Fig 1:**
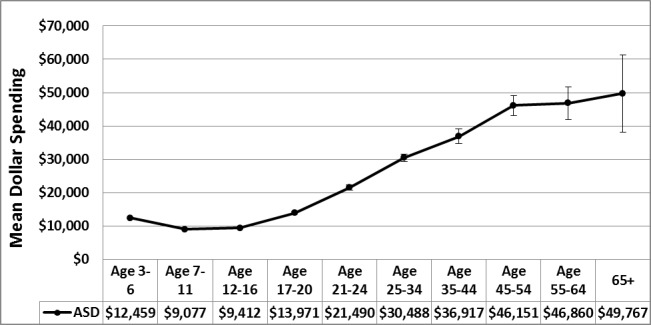
Age and mean spending for ASD. Total number of subjects for ASD = 42,274. Dots represent mean spending and thin vertical lines represent 95% confidence intervals.

CDDS spent far more on adults than on children and adolescents with ASD (Table 2). For males, the difference between the two age groups was $16,003 (p < 0.0001); spending on 18+ year old males was 152.6% above the male mean for ages 3–17. For females, the difference was $15,836 (p<0.0001); spending on 18+ year old females was 146.8% above the female mean for ages 3–17. Age differences are further highlighted in Fig 1. Annual mean spending per person at ages 3–6 was $12,459 whereas at ages 65+ annual mean spending was $49,767. Annual mean spending increased between every age group from 7–11 through 65+.

**Fig 2:**
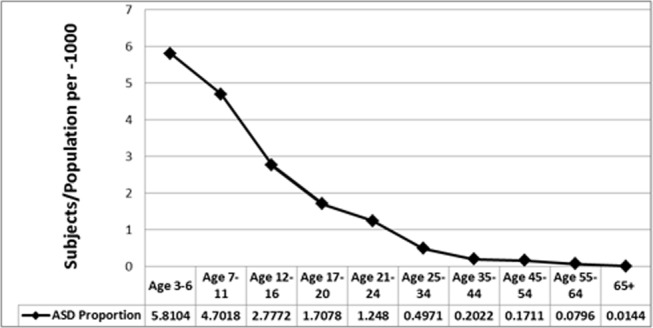
Age, number of subjects receiving services for ASD divided by population of California in same age group, per-1000. Total number of subjects for ASD– 42,274.

Fig 2 presents data on the CDDS-specific prevalence of people receiving services measured as the ratio of subjects divided by the California population in 2012, per 1000 people. Prevalence of receipt of services was highest for the youngest ages and showed a steady decline until roughly ages 45+ at which point prevalence leveled off.

Table 3 presents results on race/ethnicity for ages 3–17. Of 31,517 subjects, 12,022 (38.1%) were Hispanic, 9,175 (29.1%) were non-Hispanic white, 4,160 (13.2%) were non-Hispanic Asian, 4,136 (13.1%) were “Other”, and 2,024 (6.4%) were non-Hispanic African-American. The ranking from most to least spending was: whites, Asians, Others, Hispanics, and African-Americans. (We eschew the term “non-Hispanic” for ease of presentation.) Compared to whites, spending was $1,998 (17.4% of the white mean) lower for African-Americans, and $1,909 (16.6%) lower for Hispanics; both differences generated p-values less than 0.0001. Differences between whites on the one hand and Asians and Others on the other were small, $444 and $449, and not statistically significant. Both African-Americans and Hispanics received significantly less funding than either Asians (p<0.0001) or Others (p<0.0001).

**Table 3:** Race and ethnicity, ages 3–17, number of subjects, means and differences for spending. N = 31,517.

Categories	African-American, non-Hispanic,	Hispanic	Asian, non-Hispanic	Other and unknown	White, non-Hispanic,
Number of subjects	2,024	12,022	4,160	4,136	9,175
Mean spending	$9,482	$9,571	$11,036	$11,031	$11,480
Standard deviation of spending	$13,502	$12,643	$15,152	$13,467	$16,519
Mean differences and p-values subtracting column value minus row value					
Hispanic	$9,482				
	-$9,571				
	= -$89				
	(p = 0.7819)				
Asian non-Hispanic	$9,482	$9,571			
	-$11,036	-$11,036			
	= -$1,554	= $1,465			
	(p<0.0001)**	(p<0.0001)**			
Other	$9,482	$9,571	$11,036		
	-$11,031	-$11,031	-$11,031		
	= -$1,549	= -$1,460	= $5		
	(p<0.0001)**	(p<0.0001)**	(p = 0.9873)		
White non-Hisp.	$9,482	$9,571	$11,036	$11,031	
	-$11,480	-$11,480	-$11,480	-$11,480	
	= -$1,998	= -$1,909	= $444	= $449	
	(p<0.0001)**	(p<0.0001)**	(p = 0.1276)	(p = 0.0979)	

** Indicates statistical significance at the 0.01 level, 2-tailed test.

Somewhat different patterns were found in adults (Table 4). Of 10,757 adult subjects, 5,168 (48.0%) were whites, 2,133 (19.8%) were Hispanic, 1,263 (11.7%) were Asians, 1,237 (11.5%) were African-Americans, and 956 (8.9%) were in Others. Similar to children and adolescents, mean spending per person was highest for whites, but the ranking from greatest to least was different: whites, African-Americans, Others, Asians, and Hispanics. Compared to whites, spending was $12,970 (41.8% of the white mean) lower for Hispanics, $8,015 (25.8%) lower for Asians, $5,613 (18.1%) lower for Others, and $4,177 (13.5%) lower for African-Americans. All of the differences compared to whites garnered p-values less than 0.0001.

**Table 4:** Race and ethnicity, ages 18+, number of subjects, means and differences for spending. N = 10,757.

Categories	African-American, non-Hispanic,	Hispanic	Asian non-Hispanic	Other and unknown	White, non-Hispanic,
Number of subjects	1,237	2,133	1,263	956	5,168
Mean spending	$26,831	$18,038	$22,993	$25,395	$31,008
Standard deviation of spending	$35,020	$26,148	$29,721	$38,541	$41,498
Mean differences and p-values subtracting column value minus row value					
Hispanic	$26,831				
	-$18,038				
	= $8,793				
	(p<0.0001)**				
Asian non-Hispanic	$26,831	$18,038			
	-$22,993	-$22,993			
	= $3,838	= -$4,955			
	(p<0.0032)**	(p<0.0001)**			
Other	$26,831	$18,038	$22,993		
	-$25,395	-$25,395	-$25,395		
	= $1,436	= -$7,357	= -$2,402		
	(p<0.3681)	(p<0.0001)**	(p = 0.1096)		
White non-Hisp.	$26,831	$18,038	$22,993	$25,395	
	-$31,008	-$31,008	-$31,008	-$31,008	
	= -$4,177	= -$12,970	-$8,015	= -$5,613	
	(p<0.0001)**	(p<0.0001)**	(p<0.0001)**	(p<0.0001)**	

** Indicates statistical significance at the 0.01 level, 2-tailed test.

**Fig 3:**
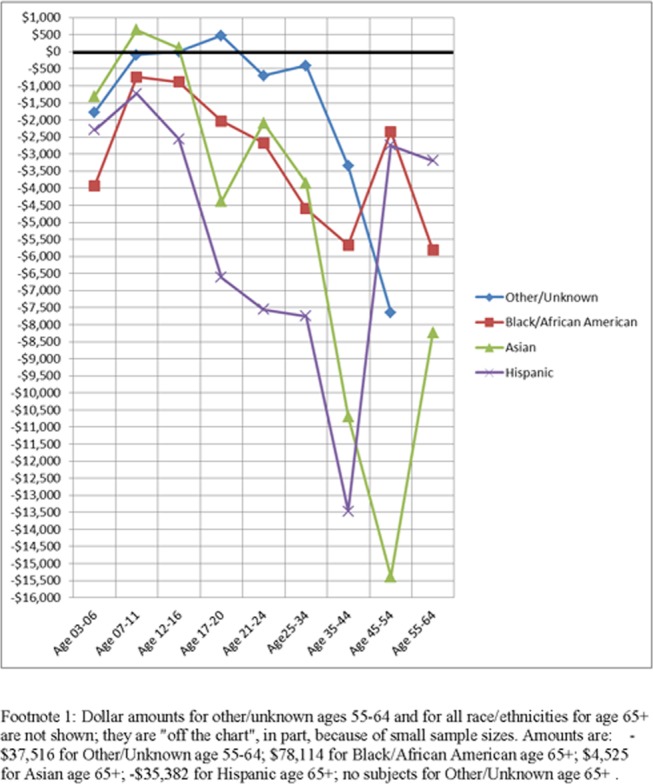
Differences in mean spending for ASD between all other race/ethnicities and whites, e.g. Hispanics–whites, stratified by age groups.

Fig 3 presents data on racial/ethnic differences across age groups. Each race/ethnicity category is compared to non-Hispanic white. That is, spending for white non-Hispanics was subtracted from the four non-white categories. For example, the first value for African-Americans age 3–6, negative $3928, resulted from subtracting $14,067 (the white non-Hispanic mean spending for ages 3–6) from $10,140 (the African-American mean spending for ages 3–6) and rounded to the nearest dollar. The lion’s share of these differences were negative, demonstrating that non-Hispanic white subjects were receiving more funds than other race and ethnicities at virtually all ages. The greatest negative values were for Hispanics. Moreover, in analyses available from the authors, all upper confidence intervals (CIs) for mean spending for Hispanics were below all lower confidence intervals for mean spending for whites, indicating strong statistical significance. In eight other comparisons, upper confidence intervals for African-Americans, Asians, and Others were below lower confidence intervals for whites. The reverse—white’s upper CIs below any other race/ethnic group’s lower CIs—never occurred.

### Two sub-samples: Persons with ASD only and Persons with ASD and ID

Our main sample was comprised of two non-overlapping sub-samples: one for persons with ASD only (n = 30,164) and another for persons with ASD and ID (ASD+ID) (n = 12,110). In the appendix we analyzed each subsample separately. (S1 Appendix).

We wanted to answer this question: Were the findings in the main sample more likely driven by the underrepresented subsample of ASD+ID or the oversampled ASD only group? Six appendix tables and one appendix figure were constructed. Two appendix tables analyzed gender differences and the two sub-samples for ages 3–17 and ages 18+ (separately); four race and ethnic tables analyzed the two age groups (separately) and the sub-samples (separately). The appendix figure displayed two line drawings—one for ASD only and another for ASD+ID—of average costs over the 10 age groups identified in Fig 1. A summary of the findings for this auxiliary analysis appear in Table 5. Findings involving gender were identical to those for the main sample, i.e. no gender differences were found within either the ASD only or ASD+ID sub-samples. Findings for race and ethnicity among the young age group (3–17), while not identical, were similar across the three samples. For example, all three had whites, Others and Asians ranked higher than Hispanics and African-Americans in per-person spending and all three reported no statistically significant differences between Hispanics and African-Americans. A different pattern was observed for persons 18+, however. The findings in the main sample for persons 18+ appeared to more closely mirror those of ASD+ID sub-sample than the ASD only sample. In both the main sample and the ASD+ID sub-sample for persons 18+, African-Americans ranked second in spending whereas in the ASD only sub-sample, they ranked fourth. Moreover, statistically significant differences were found between whites and all four non-white categories in the main sample and the ASD+ID sub-sample whereas statistically significant differences were found only between whites and Hispanics in the ASD only sub-sample.

**Table 5:** Summary of findings for per-person spending from main sample and two sub-samples^*^.

Demographic Group	Main sample (ASD only + (ASD+ID)	ASD only	ASD+ID
Gender, ages 3–17; and ages 18+	No statistically significant difference between males and females.	No statistically significant difference between males and females. 2.ASD only spending was about the same as ASD +ID spending for ages 3–17; ASD+ID spending was nearly double that of ASD only spending for age group 18+.	No statistically significant difference between males and females. 2.ASD only spending was about the same as ASD +ID spending for ages 3–17; ASD+ID spending was nearly double that of ASD only spending for age group 18+.
Race and ethnicity, ages 3–17	1.The ranking, from most spending to least was: white, Asian, Other, Hispanic, and African-American. Six of 10 comparisons were statistically significant; the four that were not were African-American versus Hispanic, Asian versus Other, Asian versus white, and Other verses white.2.Whites were paid 21.1% and 20.0% more than African-Americans and Hispanics, respectively, and these differences were statistically significant.	1.The ranking, from most spending to least was: white, Other, Asian, Hispanic, and African-American. Six of 10 comparisons were statistically significant; the four that were not were African-American versus Hispanic, Asian versus Other, Asian versus white, and Other versus white.2.Whites were paid 20.9% and 16.5% more than African-American and Hispanics, respectively, and these differences were statistically significant.	1.The ranking, from most spending to least was: white, Asian, Other, African-American, and Hispanic. Six of 10 comparisons were statistically significant. The four that were not significant included African-American versus Hispanic, African-American versus Other, Asian versus Other, and Asian versus white. 2.Whites were paid 27.9%, 37.8%%, and 16.3% more than African-Americans, Hispanics, and Others respectively, and these differences were statistically significant.
Race and ethnicity, ages 18+	1.The ranking, from most spending to least was: white, African-American, Other, Asian, and Hispanic. Eight of 10 comparisons were statistically significant; the two that were not were Other versus African-American and Other versus Asian.2.Whites were paid 15.6%, 71.9%, 34.9%, and 22.1% more than African-Americans, Hispanics, Asians, and Others, respectively, and these differences were statistically significant. 3.African-Americans were paid 48.7% more than Hispanics and the difference was statistically significant.	1.The ranking, from most spending to least was: Other, white, Asian, African-American, and Hispanic. Only 5 of 10 comparisons were statistically significant. The 5 that were not were African-American versus Asian, African-American versus white, Asian versus Other, Asian versus white, and Other versus white. 2.Whites were paid 64.3% more than Hispanics. No other comparisons with whites were statistically significant.	The ranking, from most spending to least was: white, African-American, Other, Asian, and Hispanic. All but two comparisons—between Other versus African-American and Other versus Asian—were statistically significant.2.Whites were paid 37.9%, 40.8%, 55.6%, and 89.1% more than African-Americans, Others, Asians, and Hispanics, and these differences were statistically significant.3.African-Americans were paid 37.2% and 12.9% more than Hispanics and Asians and the differences were statistically significant.
Ten age groups from 3–6 through 65+	Relatively similar spending from age 3 through 17; rapid increases beginning with age group 17–20 and for each group thereafter.	Relatively similar spending from age 3 through 17; modest increases beginning with age group 17–20 and for each group thereafter except for a dip from age group 45–54 to 55–64.	Relatively similar spending from age 3 through 17; rapid increases beginning with age group 17–20 and for each group thereafter.

*Footnote: For ease of presentation, we exclude the “non-Hispanic” designation for all non-Hispanics.

Findings across the 10 age categories appeared to partially explain the race and ethnic differences between ages 3–17 and ages 18+. For all three samples, spending was strikingly similar for ages 3–6, 7–11, and 12–16. But beginning with the 17–20 age group, the ASD+ID sub-sample findings rather than the ASD only findings appeared to more closely mirror those in the main sample. For both the main sample and the ASD+ID sub-sample, rapid increases in per-person spending began for age group 17–20 and continued thereafter for each age group through age 65+. For the ASD only sub-sample, there were modest increases beginning with age group 17–20 and for each age group thereafter with the exception of a dip from age group 45–54 to 55–64. We concluded that whereas the ASD+ID only sub-group appeared to have little to no unique contribution to the gender or race and ethnic findings among persons age 3–17 for the main sample, the ASD+ID findings appeared to be somewhat more important than the ASD only findings in explaining race and ethic differences among persons age 18+ as well as age differences among seven older age groups, 17–20 through 65+. We cannot conclude, however, that the ASD+ID sub-group was solely responsible for all the main findings regarding race and ethnic differences among the 18+ group or regarding age differences from 17–20 through 65+. Both the ASD only group and the main sample, for example, placed Hispanics last in the ranking of per-person spending for persons 18+. In addition, even though the ASD only group displayed modest increases in spending for older age categories and the main sample displayed rapid increases, both displayed increases.

### Eight expenditure categories for persons with ASD with or without ID (Main Sample)

**Fig 4:**
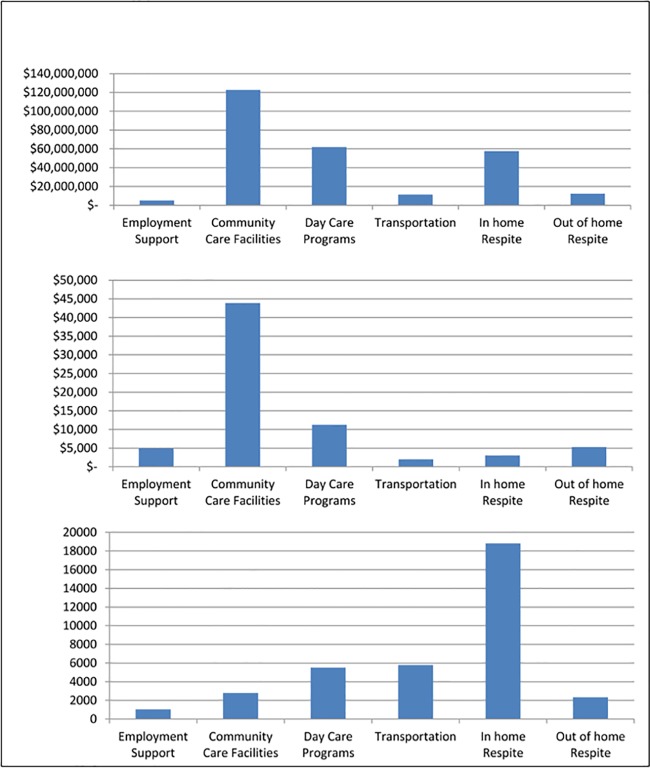
Panel A: Total Spending; Panel B: Average Spending; Panel C: Number of Recipients.

Table 6 presents data combining all ages for the eight spending categories for total spending, per-person spending and number of recipients. For total spending, from largest to smallest, the top three categories were Miscellaneous, Support Services, and Community Care Facilities. For number of recipients, the top three were Miscellaneous, In-home Respite, and Support Services. Notice that these numbers of recipients across all eight categories sum to more than the total number of recipients, 42,274, because recipients can receive more than one category of service within the year. Average spending was calculated only for those with some spending within the category. For average spending, the top three were Community Care Facilities (by far) followed by Support Services and Day Care; the bottom three were Employment Support, In-home Respite, and Transportation. Fig 4, panels A, B, and C present the same data for the more informative categories: Employment Support, Community Care Facilities, Day Care Programs, Transportation, In-home Respite, and Out-of-home Respite.

**Table 6:** Total Costs, Average Spending and Number of Recipients for all Ages Combined.

	Employment Support	Community Care Facilities	Day Care Programs	Transpor-tation	In home Respite	Out of home Respite	Support Services	Miscellaneous
**Total Spending**	$5,120,666	$122,694,671	$62,076,166	$ 11,474,622	$57,574,650	$12,327,607	$167,200,246	$167,301,205
**Average Spending**	$ 4,957	$43,867	$11,244	$ 1,981	$ 3,059	$ 5,268	$13,517	$7,450
**Number of Recipients**	1,033	2,797	5,521	5,792	18,819	2,340	12,370	22,457

**Fig 5:**
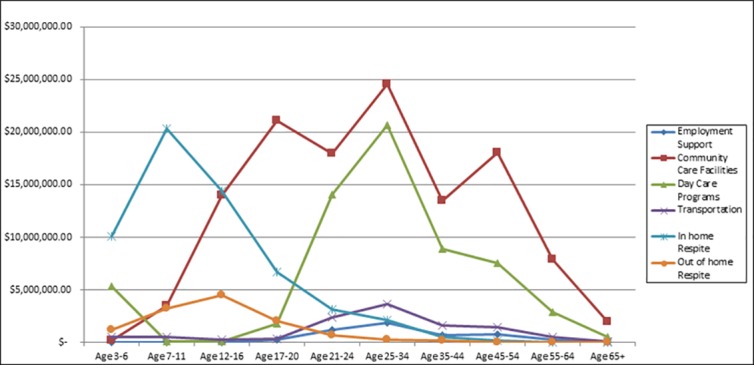
Total Spending by Age.

**Fig 6:**
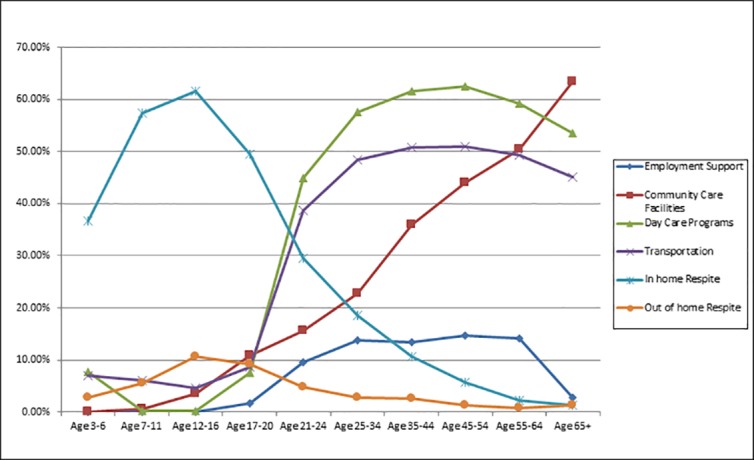
Percentage of Recipients by Age.

**Fig 7:**
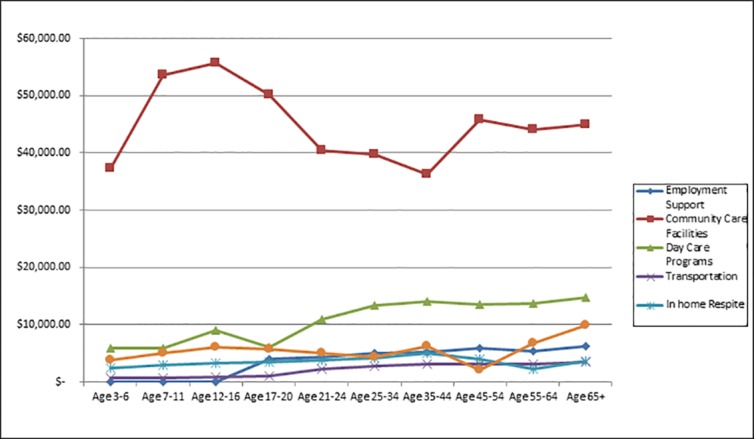
Average Spending by Age.

Figs 5–7 present line drawings for total spending, percent of recipients, and average spending across 10 age groups for the six more informative categories. Corresponding tables (Tables 7, 8 and 9) present numerical data on all eight categories. Fig 5, displaying total spending., shows that Employment Support registered zero dollars until the 17–20 age bracket, peaked for the 25–34 bracket, and fell for the older age brackets. Community Care Facilities was the top category among these six from ages12 through 65+. Transportation displayed a hill pattern and peaked at age 25–34. For the early years, ages 3–16, the bottom categories (for greatest to least) were Transportation and Day Care Programs. For the adult years, 21–65+, the lowest spending categories were In-home and Out-of-home Respite. In the corresponding table (Table 7) that includes all eight categories, the highest values (by far) were found in the earliest years, 3–6 and 7–11 and were for Miscellaneous (roughly $93 million and $46 million) and for Support Services ($32 million and $32 million). These high values for total costs likely result from the high prevalence of diagnosed ASD among young children compared to adults.

**Table 7:** Total Spending by Age.

	Employment Support	Community Care Facilities	Day Care Programs	Transportation	In home Respite	Out of home Respite	Support Services	Miscellaneous
Age 3–6	$ 0	$ 223,377	$ 5,345,101	$ 515,816	$10,078,147	$1,223,984	$31,692,102	$92,997,388
Age 7–11	$0	$3,482,867	$ 131,043	$ 517,161	$20,360,714	$3,284,907	$31,808,949	$46,091,425
Age 12–16	$0	$13,963,131	$ 127,394	$ 285,493	$14,368,216	$4,533,795	$16,374,627	$15,978,657
Age 17–20	$246,083	$21,045,757	$ 1,807,477	$ 371,232	$ 6,708,297	$2,044,289	$14,194,238	$5,870,123
Age 21–24	$1,184,305	$17,966,455	$14,054,643	$2,431,680	$3,162,653	$ 688,916	$17,989,413	$2,689,718
Age 25–34	$1,913,695	$24,555,816	$20,675,728	$3,645,168	$ 2,120,374	$ 323,833	$26,461,457	$2,125,827
Age 35–44	$ 732,294	$13,502,355	$8,938,147	$1,622,930	$548,652	$ 173,915	$11,881,315	$740,196
Age 45–54	$765,089	$18,052,261	$7,563,677	$1,443,204	$206,281	$ 23,925	$11,470,371	$433,122
Age 55–64	$ 266,735	$7,884,482	$ 2,873,800	$532,542	$17,643	$20,180	$4,618,510	$336,951
Age 65+	$12,466	$2,018,170	$559,156	$109,396	$3,672	$9,863	$709,264	$37,798

**Table 8:** Percentage by Age.

	Employment Support	Community Care Facilities	Day Care Programs	Transportation	In home Respite	Out of home Respite	Support Services	Miscellaneous
**Age 3–6**	0.00%	0.05%	7.75%	6.97%	36.69%	2.72%	28.30%	69.82%
**Age 7–11**	0.00%	0.55%	0.19%	6.03%	57.45%	5.56%	31.18%	57.03%
**Age 12–16**	0.00%	3.53%	0.20%	4.62%	61.63%	10.65%	26.37%	48.14%
**Age 17–20**	1.63%	10.84%	7.61%	8.70%	49.46%	9.22%	27.41%	39.98%
**Age 21–24**	9.54%	15.55%	44.90%	38.61%	29.49%	4.82%	31.83%	32.11%
**Age 25–34**	13.84%	22.84%	57.49%	48.49%	18.60%	2.80%	29.66%	32.35%
**Age 35–44**	13.38%	35.90%	61.50%	50.82%	10.68%	2.69%	29.16%	34.26%
**Age 45–54**	14.65%	44.07%	62.42%	50.89%	5.70%	1.34%	33.33%	36.58%
**Age 55–64**	14.09%	50.42%	59.15%	49.30%	2.25%	0.85%	29.86%	36.34%
**Age 65+**	2.82%	63.38%	53.52%	45.07%	1.41%	1.41%	26.76%	25.35%

**Table 9:** Average Spending by age.

	Employment Support	Community Care Facilities	Day Care Programs	Transportation	In home Respite	Out of home Respite	Support Services	Miscellaneous
**Age 3–6**	$ 0	$37,230	$5,926	$635	$2,359	$3,861	$9,618	$11,439
**Age 7–11**	$ 0	$53,583	$5,957	$733	$3,025	$5,046	$8,708	$6,899
**Age 12–16**	$ 0	$55,630	$9,100	$870	$3,280	$5,989	$8,738	$4,671
**Age 17–20**	$3,906	$50,109	$6,127	$1,102	$3,501	$5,726	$13,366	$3,790
**Age 21–24**	$4,338	$40,374	$10,937	$2,201	$3,747	$4,992	$19,747	$2,927
**Age 25–34**	$5,103	$39,670	$13,271	$2,774	$4,207	$4,261	$31,168	$2,296
**Age 35–44**	$5,268	$36,199	$13,988	$3,074	$4,943	$6,211	$39,212	$2,079
**Age 45–54**	$5,840	$45,818	$13,555	$3,172	$4,045	$1,994	$38,491	$1,325
**Age 55–64**	$5,335	$44,047	$13,685	$3,043	$2,205	$6,727	$43,571	$2,612
**Age 65+**	$6,233	$44,848	$14,715	$3,419	$3,672	$9,863	$37,330	$2,100

Fig 6 presents data for the percentage that received services in the specific category, among all persons with ASD who received some services, again, for the six more informative categories. For example, the 1.63% for Employment Support, ages 17–20 indicates that 1.63% of all recipients age 17–20 participated in an Employment Support program. Notice that these percentages were zero for children, as expected, given that children do not hold jobs. For adults aged 20–64, Employment Support participation was relatively low, between 9.5% to 14.7%. Across all categories but within any age group, these percentages can sum to more than 100% because recipients can obtain services from more than one category. Percentages served within Community Care Facilities rose consistently with age, beginning with 0.05% and 0.55% for ages 3–6 and 7–11 and peaking at 63.4% for ages 65+. Day Care Programs and Transportation displayed similar patterns: relatively low from ages 3–6 to ages 17–20 but rising rapidly beginning with ages 21–24, peaking at groups middle age, and declining slightly at ages 65+. Among children, Day Care participation was highest in preschool years. For In-home Respite, the greatest percentages were for the youngest ages and percentages gradually and uniformly dropped for increasing ages. Out-of-home Respite followed a slightly different pattern with relatively smaller percentages for the youngest (aged 3–6), largest percentages for older children and youth ages 7–20, then declining for every subsequent age group. In the corresponding table (Table 8) the percentages of recipients within the Support Services and Miscellaneous categories were first and third in the rankings from ages 3–6 through 17–20, but declined in the rankings for older ages. For most adult age groups, the percentage of clients participating were highest for Day Care Programs (44.9% - 62.4%) and Transportation ($38.6%- 50.9%).

Fig 7 presents data for average, per-person spending for persons who received at least one dollar of service for the six more informative categories. Average spending for Employment Support was zero until age 17 but abruptly rose to $3906 for ages 17–20 and gradually rose to over $6,000 for age 65+. For adults aged 20–64, Employment Support per-person spending was in a relatively narrow range, $4338 to $5840. Community Care Facilities average spending was, by far, the highest among all other categories, except for age group 35–44 for which Support Services had the highest spending. Community Care Facilities began below $40,000 for ages 3–6, peaked for ages 7–11 and 12–16 above $50,000 and then gradually fell to the high $30,000 and low $40,000 range for adults. Day Care Programs were in the $5,000 to $9,000 range for children and youth, but increased significantly for adults to the $13,000 to $14,000 range. Compared to other categories, Transportation was relatively lower for children and youths ($600 to $1,100) than for adults ($2,000 to $3,000). In the corresponding table (Table 9) average spending for Support Services and Miscellaneous displayed opposing patterns. Support Services began relatively low at $9,618 but gradually rose for each additional age group reaching a peak of $43,571 for age group 55–64. Miscellaneous average spending, on the other hand, began relatively high at $11,439 for ages 3–6 but then fell for most age groups until reaching a low of $1,325 for ages 45–54 and remaining low for ages 55–64 and 65+.

Recall the aggregate average spending for all categories combined across age groups in Fig 1. Except for the youngest group (3–6), aggregate average spending increased every year of age from 7–11 through 65+. Data from Fig 7 suggest that the age patterns for Employment Support, Day Care Programs, Transportation, and Support Services were the likely causes for the increase in aggregate average spending from childhood to adulthood.

## Discussion

Findings revealed far more males than females receiving services for ASD, as is well established. The difference in spending was only slightly in favor of females. The similar spending for males and females is comparable with other research[24]. Moreover, the sex-ratio data in the CDDS are comparable to other studies that show approximately four times as many males as females with ASD[24,42,43].

We found dramatic differences in the CDDS-specific prevalence of receipt of developmental services across age groups: a much higher prevalence of receipt of services for ASD was found for children compared to youths, youths compared to adults, and adults compared to seniors. These results are consistent with the surge in diagnoses of ASD among children and youths over the past 15 years but should not be confused with estimates of overall prevalence of ASD.

Per-person CDDS funding across age groups also varied strikingly with adults receiving approximately two and one-half times more than children and adolescents. There are likely several reasons for these differences. First, these age patterns are consistent with the high spending associated with supporting adults who require expensive employment training and assistance as well as residential care; affected adults are less likely than children to be cared for by parents or other relatives[4,30].^4^Second, there could be a cohort effect. The typical 55-year old may have been diagnosed in the 1960s or 1970s and have more severe ASD than the typical 25 year old who may have been diagnosed in the 1980s or 1990s. Third, the total amount of government spending for children and youths with ASD may be much higher than that solely provided by CDDS if public school funding that is not available to adults is taken into account[44].

In a related finding, annual average spending per person was higher for ages 3–6 than for either ages 7–11 or 12–16. There may be three explanations for this finding. First, differences in severity may be a factor. Children with relatively severe ASD are much more likely to be diagnosed earlier. Consequently, those with ASD in the youngest age group are more likely to have severe symptoms and require more services. Second, it could be that there are more proven successful behavioral interventions for very young autistic children than for children and youths ages 7–16. Third, as mentioned above, school-age children may be receiving services through other funding streams not available in the preschool years.

Our race/ethnicity findings can be compared to the literature. First, the percentages in each category were similar to those for the entire population within California in 2010–2011: 40% non-Hispanic white, 39% Hispanic, 15% non-Hispanic “other,” (including Asian) and 6% non-Hispanic African-American[45]. In addition, similar to our findings, previous studies have found higher prevalence among non-Hispanic whites than either non-Hispanic African-Americans or Hispanics[1,7,42].The largest differences in annual mean spending per person between the California population and CDDS subjects pertained to Other; this difference was likely explained by the possibility that CDDS data included non-responders in the Other category (CDDS did not have a separate category for non-responders).

We found higher annual mean spending per person on non-Hispanic whites than for any other race/ethnicity category and among the lowest amounts for Hispanics for all age groups and among African-American non-Hispanics for persons age 3–17. Moreover, the differences between white non-Hispanics on the one hand and African-American non-Hispanics and Hispanics on the other occurred at every age and these differences were larger for adults than for children and youths. Not many studies have examined racial/ethnic disparities in spending on ASD, but those that have done so have found spending was higher per person for white than non-white children[24–26]. Shattuck et al.[46] found that disproportionate numbers of Wisconsin Medicaid enrollees were from census areas with high percentages of white families compared to other areas in Wisconsin.

We also found standard deviations much larger than means for per-person costs. This was expected. Dollar amounts for costs are generally skewed with a long right tail resulting in large standard deviations[47].

With respect to the eight expenditure categories, we found that spending and participation varied across age groups. Total spending declined with age largely due to the decline in numbers of recipients among older age groups. These findings are consistent with the surge in diagnosed ASD over the past 15 years. Community Care Facilities, Day Care Programs, and Transportation displayed similar “hill” patterns for total spending and percentages who received services: relatively low for young ages, peaking for ages 17–20, 21–24 and 25–34, and then declining thereafter. But the decline in total spending and participation masks changes in spending per-person. Most previous studies document spending categories for children and youths[4]. Our study also documents spending for adults. Average spending for Employment Support gradually rose from ages 17–20 to ages 55–64 and 65+. Average spending amounts on Community Care Facilities were, by far, the largest of any categories for virtually every age. Average spending for Day Care Programs and Transportation were relatively low for children and youths but relatively high for adults.

There are limitations. First, our data are neither a census nor a random sample of California; the data are from persons who apply for and receive services from CDDS. The CDDS has been estimated to capture data on roughly 75–80% of all children with autism in California as some parents do not apply while other families pay out-of-pocket for behavioral services and/or receive therapies through local school districts[22,23]. For FY 2012–2013, 42,274 California residents received services for ASD out of a resident population of approximately 38 million. The CDDS data likely over-represent relatively severe cases. People with mild ASD may not apply for CDDS help or they may not be eligible for CDDS services for lack of sufficient severity of disabilities.

A second limitation is that California may not be representative of the United States; nevertheless, California is the most populous state. Moreover, numerous studies using CDDS data have been used to assess epidemiologic and economic issues surrounding autism for the entire nation[4,12,13,16–28].An advantage of this database was the racial/ethnic diversity of the state, which permitted robust comparisons involving Hispanics, non-Hispanic African-Americans, and non-Hispanic Asians.

Another possible limitation is our definition of who qualifies for the ASD group. Following other authors[4,27,30,34,39] we combined ASD only with ASD plus ID in our main analysis. But in an auxiliary analysis we analyzed sub-samples for ASD only and ASD+ID. Our main findings, by and large, were confirmed in both sub-samples. We nevertheless did find that the ASD+ID sub-sample figured more prominently in our main sample findings for adults than did the sub-sample of persons with ASD only.

The explanation for the low percentage of persons in the ASD plus ID sub-sample in the CDDS is likely due to the fact that administrative diagnoses of ID are relatively less commonly recorded than the true prevalence of ID[48]. Peacock et al.[24] reported that just 16% of Medicaid-enrolled children with ASD diagnoses also had ID diagnoses recorded. Nevertheless, the reported prevalence of ID in ASD has been decreasing over time[49] for many reasons, including ascertainment bias[50], poorer identification of milder forms of ASD in early studies[51], and the effectiveness of early intervention on autism symptoms[52–54].

A related possible limitation pertains to epilepsy. Approximately one-half of one percent of CDDS subjects with ASD were also recorded with either cerebral palsy or epilepsy and this one-half was excluded from our analysis. It is likely that CDDS data significantly underestimate co-occurrence of ASD with epilepsy[40]. But the same CDDS recording phenomena that apply to ID likely also apply to epilepsy. CDDS only requires one recorded condition to receive benefits. Parents or adults with conditions may find it less stigmatizing to record ASD rather than epilepsy. This argument suggests that we may have some persons with both ASD and epilepsy in our sample.

Additional limitations involve the scope of the data. CDDS does not capture all spending on services for individuals with ASD or ID, in part because not all California residents with ASD or ID receive services through CDDS. In addition, significant amounts are spent on medical care by employer-provided health insurance, private insurance, Medicaid, and Medicare[43,55,56].Significant lost-wage income, food and housing support amounts are spent by the Social Security Disability Insurance and Supplemental Income programs, federal and state Earned Income Tax Credit programs, the federal food stamp program, and the Temporary Assistance to Needy Families program[55]. Moreover, individuals and families contribute substantial amounts in out-of-pocket expenses[30,34,39].

A different limitation involves the age of the data. Upon publication of this paper, CDDS will have more recent data. Our analysis of the 2012–2013 data can nevertheless be used as a baseline to measure progress in future years.

Final limitations concern family income. First, CDDS does not collect data on family income. It could be that the racial disparities observed in these data reflect the low income of minorities rather than purely racial or ethnic differences. Second, CDDS collects fees for its services that are based upon a sliding scale of income. The first fee is for families of persons who reside in their own or family home; the second, for families of persons who reside in a Community Care or other facility (Personal telephone communication with Dean Shellenberger, Manager of Regional Fee Program, California Department of Developmental Services, Sacramento, California, December 4, 2015). The first fee is modest, at most $200 annually, and applies to all families of all persons <18 years old[57]. The second fee can be substantial. For example, consider a family of four with one child. Up though $20,000 per year, the family has a $0 monthly fee in 2015. For incomes $20,001 to $25,000 the monthly fee is $60. For income over $175,000, the monthly fee is $1770. But this second fee is assessed only to a small minority: 1–8% of CDDS clients age <18 reside in facilities. Moreover, this fee is not assessed to clients > = 18 years of age[57]. Nevertheless, if a wealthy white family receives more dollar benefits than a poor black family, it could be that the net benefits (benefits minus fees) could narrow or eliminate the disparity. However, as far as we are aware, nothing in CDDS guidelines or provisions links dollar benefits (as opposed to fees) to family income.

Quantitative estimates are the foundation for the national effort to lower costs and improve quality of health care. ASD is an unusual condition because a large proportion of spending for care is non-medical. This study’s findings of very high non-medical annual mean spending per person in adults suggest that costs of care could rise dramatically over time, as the growing numbers of children diagnosed with ASD age into adulthood and the spending per person increases with age[58]. Understanding the service needs of adults with ASD, ways to assist them in living independently and productively, and what types of policies can simultaneously reduce the costs of the programs that are necessary and beneficial to this population are critical at this time, and rapidly becoming an urgent imperative. The issue of racial/ethnic disparities in access to and use of services by individuals with ASD is critical to address, both in California, where the majority of the population is non-white and/or Hispanic, and elsewhere. Quantification of gaps in use of services and spending on publicly-funded care for individuals with ASD who have other social and economic disadvantages highlights the problem of inequities and provides clear evidence to public officials demonstrating that current patterns of care may be reinforcing rather than countering preexisting health disparities by race and ethnicity. Our quantification of use of services across age span suggests that as the current large cohorts of children and youths become adults there will be significant increases in need for Employment Support, Community Care Facilities and Transportation services.

## Supporting Information

S1 Appendix (DOCX)

S1 Dataset (XLSX)

S2 Dataset (XLSX)
