# Supplementary material for: Spending by California’s Department of Developmental Services for Persons with Autism across Demographic and Expenditure Categories
Source: PLoS One. 2016 Mar 25;11(3):e0151970. doi: 10.1371/journal.pone.0151970 (PMC4807877; doi:10.1371/journal.pone.0151970)
Supplement: S1 Appendix — (DOCX) [file pone.0151970.s001.docx]

Appendix to “Spending by California’s Department of Developmental Services for persons with autism across demographic and expenditure categories”

Appendix Table 1 presents data comparing gender differences for ages 3-17 between the two sub-samples (ASD only and ASD+ID). Table 2 presents similar data for ages 18+. No statistically significant gender differences for average per-person spending were observed within either of the sub-samples. When comparing across sub-samples, for the 18+ age group we found that per-person spending within the ASD+ID group was nearly double the spending within the ASD only group and that these differences were statistically significant; we found no statistically significant difference across sub-samples within the 3-17 age group.

Appendix Table 1, Gender, number of subjects, means and differences for spending. ASD only and ASD plus ID, ages 3-17, N = 31,517

| Categories | Males, ASD only | Males,  ASD and ID | Females, ASD only | Females, ASD and ID |
| --- | --- | --- | --- | --- |
| Number of subjects | 21,039 | 5,135 | 4,076 | 1,267 |
| Mean spending | $10,360 | $10,807 | $10,627 | $11,318 |
| Standard deviation | $13,735 | $15,776 | $14,372 | $18,477 |
| Dollar differences and p-values for hypothesis tests subtracting column value minus row value |  |  |  |  |
| Males,  ASD and ID | -$447  (p=0.0622) |  |  |  |
| Females, ASD only | -$267 (p=0.2743) | +$180 (p=0.5675) |  |  |
| Females, ASD and ID | -$958 (p=0.0694) | -$511 (p=0.3648) | -$691 (p=0.2220) |  |

Footnotes: * Indicates significance at the 0.05 level, 2-tailed test. ** Indicates significance at the 0.01 level, 2-tailed test

Findings:1. No differences between men and women for either ASD only or ASD+ID.

2. ASD only spending per-person is about the same as ASD +ID spending.

Appendix Table 2. Gender, ASD only and ASD plus ID, number of subjects, means and differences for spending ages 18+ N = 10,757 (adding with ASD only above yields 31,517 + 10,757 = 42,274 which is exactly the correct size for the total)

| Categories | Males, ASD only | Males,  ASD and ID | Females, ASD only | Females, ASD and ID |
| --- | --- | --- | --- | --- |
| Number of subjects | 4272 | 4486 | 777 | 1222 |
| Mean spending | $17,467 | $35,084 | $17,135 | $32,663 |
| Standard deviation | $27,605 | $42,441 | $23,854 | $41,581 |
| Dollar differences and p-values for hypothesis tests subtracting column value minus row value |  |  |  |  |
| Males,  ASD and ID | -$17,617  (p<0.0001)** |  |  |  |
| Females, ASD only | +$332 (p=0.7279) | +$17,949 (p<0.0001) ** |  |  |
| Females, ASD and ID | -$15,196 (p<0.0001)** | $2421 (p=0.0724) | -$15,528 (p<0.0001) ** |  |

Footnotes: * Indicates significance at the 0.05 level, 2-tailed test. ** Indicates significance at the 0.01 level, 2-tailed test

Findings:1. No differences between men and women for either ASD only or ASD+ID.

2. ASD only is much less expensive than ASD +ID.

Race and ethnicity data appear in Appendix Tables 3-6. Tables 3 and 4 provide data for the sub-sample with persons with ASD only ; Appendix Tables 5 and 6 provide data for the sub-sample persons with ASD+ID.

Appendix Table 3: Race and ethnicity, ASD only, ages 3-17, number of subjects, means, standard deviations, and differences for spending. N = 25,115

| Categories | African-American, non-Hispanic | Hispanic | Asian, non-Hispanic | Other, non-Hispanic | White, non-Hispanic |
| --- | --- | --- | --- | --- | --- |
| Number of subjects | 1515 | 9456 | 3160 | 3271 | 7713 |
| Mean costs | $9265 | $9618 | $10,730 | $11,004 | $11,202 |
| Standard deviation | $12,515 | $12,400 | $13,194 | $13,273 | $16,021 |
| Dollar differences and p-values for hypothesis tests subtracting column value minus row value |  |  |  |  |  |
| Hispanic | $9265 - $9618 = -$353 (p=0.3075) |  |  |  |  |
| Asian, non-Hispanic | $9265 - $10,730 = -$1465 (p=0.0002)** | $9618 - $10,730 = -$1112  (p<0.0001)** |  |  |  |
| Other, non-Hispanic | $9265 - $11,004 = -$1739 (p<0.0001)** | $9618- $11,004 = -$1386  (p<0.0001)** | $10,730 - $11,004 = -$274 (p=0.4065) |  |  |
| white, non-Hispanic | $9265 - $11,202 = -$1937 (p<0.0001)** | $9618 - $11,202 = $1584 (p<0.001)** | $10,730 - $11,202 = -$472 (p=0.1123) | $11,004 - $11,202 = -$198 (p=0.5024) |  |

Footnotes

* Indicates significance at the 0.05 level, 2-tailed test. ** Indicates significance at the 0.01 level, 2-tailed test

Main Findings:

1. The ranking, from most spending to least was : white non-Hispanic, Other non-Hispanic, Asian non-Hispanic, Hispanic, African-American non-Hispanic. Six of 10 comparisons were statistically significant. The four that were not significant were African-American versus Hispanic, Asian versus Other, Asian versus white, and Other versus white.
2. White non-Hispanics were paid 20.9% (=11,202/9265 – 1) and 16.5% (11,202/9618 – 1) more than African-American non-Hispanics and Hispanics, respectively, and these differences were statistically significant. There were no statistically significant differences among Asian non-Hispanics, Other non-Hispanics and white non-Hispanics.

Additional Findings:

1. Asian non-Hispanic and Other non-Hispanic were paid 15.8% (=10,730/9265 – 1), 11.6% (=10,730/9618 – 1), 18.8% (=11,004/9265 – 1), and 14.4% (=11,004/9618 – 1) more than African-American non-Hispanics and Hispanics, respectively, and these differences were statistically significant.
2. African-American non-Hispanics and Hispanics were paid approximately the same and the difference was not statistically significant.

Appendix Table 4: Race and ethnicity, ASD only, ages 18+, number of subjects, means, standard deviations, and differences for spending. N = 5049

| Categories | African-American, non-Hispanic | Hispanic | Asian, non-Hispanic | Other, non-Hispanic | White, non-Hispanic |
| --- | --- | --- | --- | --- | --- |
| Number of subjects | 402 | 920 | 585 | 519 | 2623 |
| Mean costs | $17,061 | $11,512 | $17,261 | $20,772 | $18,911 |
| Standard deviation | $19,484 | $16,322 | $28,959 | $35,540 | $28,377 |
| Dollar differences and p-values for hypothesis tests subtracting column value minus row value |  |  |  |  |  |
| Hispanic | $17,061 - $11,512 = $5549 (p<0.0001)** |  |  |  |  |
| Asian, non-Hispanic | $17,061 - $17,261 = -$200 (p=0.8968) | $11,512 - $17,261 = -$5749 (p<0.0001)** |  |  |  |
| Other, non-Hispanic | $17,061 - $20,772 = -$3711 (p=0.0435)* | $11,512 - $20,772 = -$9260 (p<0.0001)** | $17,261 - $20,772 = -$3511 (p=0.0742) |  |  |
| white, non-Hispanic | $17,061 - $18,911 = -$1850 (p=0.0982) | $11,512 - $18,911 = -$7399 (p<0.0001)** | $17,261 - $18,911 = -$1650 (p=0.2110) | $20,772 - $18,911 = $1861 (p=0.2610) |  |

Footnotes

* Indicates significance at the 0.05 level, 2-tailed test

** Indicates significance at the 0.01 level, 2-tailed test

Main Findings:

1. The ranking, from most spending to least was : Other non-Hispanic, white non-Hispanic, Asian non-Hispanic, African-American non-Hispanic, and Hispanic.
2. Whites were paid 64.3% more than Hispanics. No other comparisons with whites were statistically significant.

Additional findings:

1. Hispanics were paid 32.5% (=1 - 11,512/17,061), 33.3% (=1 - 11,512/17,261 ) , 44.6% (=1 - 11,512/20,772/) , and 39.1% (=1 - 11,512/18,911) less than African-American non-Hispanics, Asian non-Hispanics, Other non-Hispanics, and white non-Hispanics, respectively, and all of these differences were statistically significant.
2. The only additional statistically significant result was this : Other non-Hispanics were paid 21.8% (=20,772/17,061 – 1) more than white non-Hispanics.

Appendix Table 5: Race and ethnicity, ASD + ID, ages 3-17, number of subjects, means, standard deviations, and differences for spending. N = 6402

| Categories | African-American, non-Hispanic | Hispanic | Asian, non-Hispanic | Other, non-Hispanic | White, non-Hispanic |
| --- | --- | --- | --- | --- | --- |
| Number of subjects | 509 | 2566 | 1000 | 865 | 1462 |
| Mean costs | $10,127 | $9397 | $12,005 | $11,134 | $12,949 |
| Standard deviation | $16,082 | $13,503 | $20,102 | $14,183 | $18,869 |
| Dollar differences and p-values for hypothesis tests subtracting column value minus row value |  |  |  |  |  |
| Hispanic | $10,127 - $9397= $730 (p=0.3375) |  |  |  |  |
| Asian, non-Hispanic | $10,127 - $12,005 = -$1878 (p=0.0493)* | $9397 = -$12,005 = -$2608  (p=0.0002)* |  |  |  |
| Other, non-Hispanic | $10,127 - $11,134 = -$1007 (p=0.2420) | $9397 - $11,134= -$1737  (p=0.0016)* | $12,005- $11,134 = $871 (p=0.2750) |  |  |
| white, non-Hispanic | $10,127 - $12,949 = -$2822 (p=0.0011)* | $9397 - $12,949 = -$3552 (p<0.0001)** | $12,005 - $12,949 = -$944 (p=0.2408) | $11,134 - $12,949 = -$1815 (p=0.0085)* |  |

Footnotes

* Indicates significance at the 0.05 level, 2-tailed test. ** Indicates significance at the 0.01 level, 2-tailed test

Main Findings:

1. The ranking, from most spending to least was : white non-Hispanic, , Asian non-Hispanic, Other non-Hispanic , African-American non-Hispanic, Hispanic. Six of 10 comparisons were statistically significant. The four that were not significant included African-American versus Hispanic, African-American versus Other, Asian versus Other , and Asian versus white.
2. White non-Hispanics were paid 27.9% (=12,949/10,127 – 1) , 37.8%% (=12,949/9397 – 1) , and 16.3% (=12,949/11,134 – 1) more than African-American non-Hispanics , Hispanics, and Other non-Hispanics, respectively, and these differences were statistically significant.

Additional Findings:

1. Asian non-Hispanics were paid 18.5% (=12,005/10,127 – 1) and 27.8% (=12,005/9397 – 1) more than African-American non-Hispanics and Hispanics, respectively, and these differences were statistically significant.
2. African-American non-Hispanics and Hispanics were paid approximately the same and the difference was not statistically significant.

Appendix Table 6: Race and ethnicity, ASD + ID, ages 18+, number of subjects, means, standard deviations, and differences for spending. N = 5708

| Categories | African-American, non-Hispanic | Hispanic | Asian, non-Hispanic | Other, non-Hispanic | White, non-Hispanic |
| --- | --- | --- | --- | --- | --- |
| Number of subjects | 835 | 1213 | 678 | 437 | 2,545 |
| Mean costs | $31,535 | $22,987 | $27,940 | $30,886 | $43,476 |
| Standard deviation | $39,583 | $30,722 | $29,503 | $41,200 | $48,593 |
| Dollar differences and p-values for hypothesis tests subtracting column value minus row value |  |  |  |  |  |
| Hispanic | $31,535 - $22,987 = $8548 (p<0.0001)** |  |  |  |  |
| Asian, non-Hispanic | $31,535 - $27,940 = $3595 (p=0.0431)* | $22,987 - $27,940 = -$4953 (p=0.0006)** |  |  |  |
| Other, non-Hispanic | $31,535 - $30,886 = $649 (p=0.7869) | $22,987 - $30,886 = -$7,899 (p=0.0003)** | $27,940 - $30,886 = -$2,946 (p=0.2163) |  |  |
| white, non-Hispanic | $31,535 - $43,476 = -$11,941 (p<0.0001)** | $22,987 - $43,476 = -$20,489 (p<0.0001)** | $27,940 - $43,776 = -$15,536 (p<0.0001)** | $30,886 - $43,476 = -$12,590 (p<0.0001)** |  |

Footnotes

* Indicates significance at the 0.05 level, 2-tailed test. ** Indicates significance at the 0.01 level, 2-tailed test

Findings:

1. The ranking, from most spending to least was: white non-Hispanic, African-American non-Hispanic, Other non-Hispanic , Asian non-Hispanic, Hispanic. Moreover, all but two comparisons ---between Other and African-American and Other and Asian--- were statistically significant.
2. White non-Hispanics were paid 37.9% (=43,476/31,535 – 1) , 40.8 % (=43,476/30,886 – 1), 55.6% (=43,476/27,940 - 1), and 89.1% (=43,476/22,987 – 1) more than African-American non-Hispanics , Other non-Hispanic , Asian non-Hispanic, and Hispanic , and these differences were statistically significant.
3. African-American non-Hispanics were paid 37.2% (=31,535/22,987 -1 ) and 12.9% (=31,535/27,940 -1) more than Hispanics and Asian non-Hispanics and the differences were statistically significant.

We next compare the sub-samples for ASD only versus ASD+ID across the 10 age brackets (Appendix Figure 1) and also compare with Figure 1 in the text. Appendix Figure 1 demonstrates that per-person spending is generally flat from years 3 through 17 but begins to increase virtually every year after age 17 for both ASD only and ASD+ID. The increases are steeper for the ASD+ID sub-sample. Comparing to Figure 1 in the text is revealing. The ASD+ID sub-sample appears to more closely mirror the line drawing in Figure 1 as both rise more rapidly than the ASD only line and both rise from the 45-54 age group to the 55-64 age group whereas the sub-sample for ASD only drops slightly for these age groups.

Appendix Figure 1
